# High-level over-expression, purification, and crystallization of a novel phospholipase C/sphingomyelinase from *Pseudomonas aeruginosa*

**DOI:** 10.1016/j.pep.2012.11.005

**Published:** 2013-07

**Authors:** Daphné Truan, Adriana Vasil, Martin Stonehouse, Michael L. Vasil, Ehmke Pohl

**Affiliations:** a Swiss Light Source, Paul Scherrer Institute, 5232 Villigen, Switzerland; b Department of Microbiology, University of Colorado, School of Medicine, Anschutz Medical Center, Aurora, CO 80045, USA; c Department of Chemistry & School of Biological and Biomedical Sciences, Durham University, Durham DH1 3LE, UK

**Keywords:** *Pseudomonas aeruginosa*, Phospholipase C, Sphingomyelinase, Crystallization, l-Selenomethionine substitution, Micro-crystal

## Abstract

The hemolytic phospholipase C/sphingomyelinase PlcH from the opportunistic pathogen *Pseudomonas aeruginosa* represents the founding member of a growing family of virulence factors identified in a wide range of bacterial and fungal pathogens. In *P. aeruginosa* PlcH is co-expressed with a 17 kDa chaperone (PlcR2) and secreted as a fully folded heterodimer (PlcHR2) of approximately 95 kDa, by the twin arginine translocase (TAT) via the cytoplasmic membrane and through the outer membrane, by the Xcp (TypeII) secretory system. PlcHR2 has been shown to be an important virulence factor in model *P. aeruginosa* infections and is selectively cytotoxic, at picomolar concentrations to mammalian endothelial cells. Here we report how the various challenges starting from protein overexpression in the native organism *P. aeruginosa*, the use of detergents in the crystallization and data collection using the most advanced μ-focus synchrotron beam lines were overcome. Native diffraction data of this heterodimeric protein complex were collected up to a resolution of 4 Å, whereas needle-shaped crystals of l-selenomethionine substituted PlcHR2 with a maximum diameter of 10 micron were used to collect data sets with a maximum resolution of 2.75 Å.

## Highlights

► Overexpression of the secreted phospholipase C/sphingomyelinase PlcHR2 from *P. aeruginosa* in native and Se-methionine substituted form. ► Purification and crystallisation of PlcHR2. ► Crystalographic analysis and data collection of PlcHR2 micro-crystals.

## Introduction

*Pseudomonas aeruginosa* is an opportunistic bacterial pathogen that poses a lethal threat to patients with open injuries and burn wounds, immuno-compromised patients and above all, cystic fibrosis sufferers [1–3]. During infection the pathogen produces and secrets a variety of virulence factors including Exotoxin A, and at least four different phospholipases C (PLC) [4,5] including the recently discovered *P. aeruginosa* phospholipases PlcA and PlcB that belong to the well-characterized family of Zn-dependent PLCs [4]. The biological functions of these PLCs however, are not well understood, although PlcB has been shown to be required in the chemotaxis of *P. aeruginosa* towards phospholipids (e.g. phosphatidylcholine a major component of lung surfactant) [4,6]. Two extracellular PLCs of *P. aeruginosa* were identified earlier [7]. These are the hemolytic phospholipase C (PlcH) and its closely related non-hemolytic ortholog PlcN, which are almost twice as large as PlcA and PlcB, respectively, and show no significant sequence similarity to the Zn-dependent class of PLCs [5,8]. PlcH is co-expressed with two *in-phase* overlapping genes *plcR1*/*plcR2* that are located downstream to the *plcH* gene. The 23 KDa PlcR1 and the 17 kDa PlcR2 are believed to act as co-chaperones in the secretion of PlcH by forming heterodimeric PlcHR1 and PlcHR2 complexes [9]. These complexes are secreted into the extracellular environment in a folded state via the twin-arginine translocation (TAT) pathway through the inner membrane [10,11] and subsequently through the lipopolysaccharide containing outer membrane of *P. aeruginosa* via the Xcp (Type II) system [5].

More recently it has become clear that PlcH is the prototype of a growing superfamily of enzymes, which include a range of extracellular toxins with phospholipase C and sphingomyelinase activities. Consequently, PlcH does not merely represent a single extracellular virulence factor produced by only a single opportunistic pathogen. In fact, some fungal and numerous bacterial pathogens express one or more multiple orthologs. For example, *Aspergillus fumigatus* caries one PlcH ortholog [12], individual *Burkholderia pseudomallei* possess three genes encoding PlcH orthologs [13] and some *Mycobacterium tuberculosis* strains express as many as 4 different orthologs [14]. The emerging opportunistic pathogen *Acinetobacter baumannii*, as well as the Select Agent *Burkholderia mallei* each carry two genes encoding members of PlcH superfamily [15]. Finally, there is one member of this superfamily with a known crystal structure, AcpA from *Francisella tularensis*, which, based on sequence comparison would be situated just at the evolutionary border between known PLC/sphingomyelinase members and those enzymes, which only have phosphatase activity [16]. AcpA, which shares approximately 22% sequence identity with the catalytic domain of PlcH is a periplasmic protein with acid phosphatase activity that can utilize an assortment of biologically important phosphorylated compounds (e.g. ATP and tyrosine-PO_4_) [17].

The PlcHR2 complex secreted by *P. aeruginosa* has profound biological functions as summarized below. First of all, the enzyme has been shown to be highly active on sphingomyelin, as well as phosphatidylcholine, but it is much less active on other phospholipids that do not contain choline (e.g. phosphatidylethanolamine, phosphatidylglycerol), and it is not active on phosphatidylserine [18]. Accordingly PlcHR2 catalyzes the hydrolysis of sphingomyelin to phosphocholine and ceramide, as well as it hydrolyzes phosphatidylcholine to phosphocholine and diacylglycerol (DAG) [19]. Ceramide and DAG are important eukaryotic secondary messenger molecules implicated in a wide array of functions ranging from inflammation to apoptosis [20], but remarkably they exert very distinct effects. For example, generation of ceramide from sphingomyelin is pro-apoptotic while increased production of DAG induces a proliferative or transformation response in eukaryotic cells. It is important to note in this regard that this enzyme exhibits a highly selective cytotoxicity to human endothelial cells [12].

The three-dimensional structure of PlcHR2 will help to decipher the catalytic mechanism and hence inform about the molecular basis of enzymatic activity and endothelial cell interactions (e.g. binding to integrin receptors). Crystallographic studies on PlcHR2, however, are hampered by a multitude of difficulties on several levels. To start with, *Escherichia coli* has a significantly lower G + C content than *P. aeruginosa* (50% vs 67%) and it lacks a functional Xcp secretory system [21]. Accordingly, the PlcHR2 complex is much more efficiently translated and the heterodimeric complex is secreted as soluble protein into the extracellular compartment (i.e. culture supernatant), using the native *P. aeruginosa* expression system. Moreover, although PlcHR2 itself is not a membrane protein its substrates are membrane associated, hence the protein shows a high degree of affinity for phospholipids and membrane structures further exacerbating purification as well as crystallization. Finally, l-selenomethionine substituted protein had to be produced in order to solve the crystallographic phase problem.

Here we describe the methods we developed to overcome the intrinsic problems towards obtaining diffracting crystals. These include: (i) the optimization of protein preparation, (ii) the development of an over-expression system in a methionine auxotroph to produce l-selenomethionine substituted protein samples and (iii) the use of additives and detergents for crystal optimization. Finally, we present the usage of the μ-focus beam line X06SA at the Swiss Light Source (SLS) that enabled us to collect a complete diffraction data set to 2.75 Å resolution using crystals with dimensions of less than 10 μm. The strategies and methods we describe here are applicable to the challenges protein crystallographers face today and in the future.

## Materials and methods

### Protein expression in *P. aeruginosa*, purification and characterization

Our efforts were focused on the native PlcHR2 protein complex as the most stable and soluble complex [19]. Due to the fact that the protein failed to localize extracellularly in recombinant *E. coli* expression systems, we resorted to expression in the environment of its natural host as previously described [8]. Briefly, the protein was overexpressed in *P. aeruginosa* strain PA01 derivative ADD1976 carrying a chromosomal T7 polymerase gene under the control of the *lacUV5* promoter. The expression of *plcHR2* is controlled by the T7 promotor on the pADD3268 vector. *P. aeruginosa* was grown at 37 °C in minimal M9 media to an optical density of OD_590_ ≈ 0.6, induced with isopropylthio-β-galactopyranoside (IPTG) and then kept at 32 °C for an additional 12 h during aeration of the culture. The use of 32 °C is based on the temperature typically used for optimal expression of extracellular factors of *P. aeruginosa* [22] and hence no increase of protein yield was observed at 30 °C or 37 °C (unpublished results). Cells were first separated from the supernatant by centrifugation at 10,000*g*. Then 1.5 times volume of cold ddH_2_O was added to the supernatant containing PlcHR2 in order to reduce ionic strength before applying the solution to micro granular anion exchanger diethylaminoethyl cellulose DE52. The protein was eluted with 500 mM NaCl and after further concentration and dialysis applied to a BioRad Model 491 prep cell which was used to separate culture supernatant proteins by continuous elution native gel electrophoresis (7.5% non-denaturing polyacrylamide). The prep cell was run at constant power of 12 W, and protein fractions were eluted at pH 7.2 with a flow rate of 0.35 ml/min. Separated proteins were delivered to a fraction collector and pooled in 2 ml fractions. Peak fractions were pooled, concentrated and frozen at −80 °C. All purification procedures were carried out at 4 °C. Successive matrix-assisted laser desorption ionization (MALDI) and electron spray mass spectrometric analysis (ESI-MS) confirmed the identity of the heterodimeric PlcHR2 complex as reported earlier [8]. The overall yield was improved to 1.6 mg/l culture by increasing the aeration after IPTG induction.

### l-Selenomethionine substituted protein

The *plcHR2* genes were expressed in the same native *P. aeruginosa* T7 expression system [8] with the important addition that the *metZ* gene in *P. aeruginosa* PAO1 ADD1976 was mutated using ethylmethansulfonate (EMS). Thus, this strain is no longer capable of l-methionine biosynthesis. The mutation (T**G**G to T**A**G leading to TrpSTOP) in the *metZ* gene was verified by sequencing of PCR products from the amplified gene in this strain. For l-selenomethionine substituted protein production, the *plcHR2* genes were expressed in minimal media with l-selenomethionine at 50 μg/ml. The protein was purified using the same protocols described above. The yield was significantly lower compared to the native protein with approximately 0.6 mg/l culture.

### Crystallization of the native PlcHR2

Initial crystallization experiments were performed with native PlcHR2 in various buffer compositions and concentrations, however the most promising initial results were obtained with the protein at 1.5 mg/ml in 50 mM Tris pH 7.4, 50 mM NaCl, 0.1 mM TCEP. Early crystallization screens using standard manual vapor diffusion setup and commercially available screens [23] led to two crystal morphologies. The first crystals were obtained with 10% dioxane, 0.1 M MES buffer, pH 6.5 and 1.65 M ammonium sulfate (AS). The second crystal form was obtained with 30% PEG3350, 70 mM BisTris, pH 6.5 and 0.45 AS. Crystallization optimization strategies ranged from different experimental set-ups including hanging and sitting drops at different temperatures, batch crystallization, to the addition of commercially available and *in-house* additive screens. Tungstate and vanadate were used as these anions have been successfully used in crystallization where they occupy phosphate sites [24]. Because PlcHR2 has a high affinity for membranes (i.e. phospholipids) and therefore behaves like a membrane associated protein, the membrane protein additive screen was used to improve crystal reproducibility and quality [25]. The best native crystals were obtained with the protein concentrated to approximately 9 mg/ml in sitting drop vapor diffusion experiments with a reservoir solution of 10% dioxane, 0.1 M MES, pH 6.75 and 1.5 M AS. The drop was setup with 2 μl protein solution and mixed with 0.7 μl reservoir solution and 0.3 μl 300 mM zwittergent.

### l-Selenomethioine substituted PlcHR2

Initially crystallization was attempted using conditions close to those that were successful with the native PlcHR2 sample, however, no crystals were obtained with the dioxane containing solution. The condition based on PEG3350 led to a large number of very small crystals that typically showed multiple diffraction patterns to a maximum resolution of approximately 5 Å. In order to deal with the extensive nucleation observed in many drops various seeding techniques were employed [26]. The best crystals were obtained with a protein solution at 9 mg/ml in 50 mM Tris pH 7.4, 50 mM NaCl, 0.1 mM TCEP filtered through a 0.22 μm filter and centrifuged for ten minutes. The crucial step in reproducibly obtaining crystals was streak-seeding using a cat-whisker from micro-crystals obtained under similar conditions.

### Diffraction experiments

The first native crystals were tested at the EMBL Hamburg outstation wiggler beam line BW7B [27] located at the 2nd generation synchrotron DORIS at DESY. All consecutive diffraction experiments were performed at the 3rd generation synchrotron, the Swiss Light Source (SLS). Experiments were performed either on beam line X10SA which features a focused beam size of 50 × 10 μm [28,29] or in case of l-selenomethionine substituted PlcHR2 on the μ-focus beam line X06SA. This beam line is equipped with the high-precision microdiffractometer MD2 and allows a focus of 25 × 6 μm [30]. The small beam focus matches the size of these protein crystals much better, which significantly increases signal-to-background ratio.

Native protein crystals were typically fished directly from the drop using standard nylon loops and frozen in the cold nitrogen stream [31]. l-selenomethionine substituted crystals were extremely fragile and hence mounting only succeeded using MiTeGen MicroLoops E which also showed the lowest background on the diffraction pattern [32]. All diffraction data were collected using the rotation methods with crystals cooled to 100 K.

### Indexing, integration and scaling

All diffraction data were carefully indexed, integrated and scaled using XDS [33] and/or HKL2000 [34].

## Results and discussion

### High-yield expression and purification

**Fig. 1:**
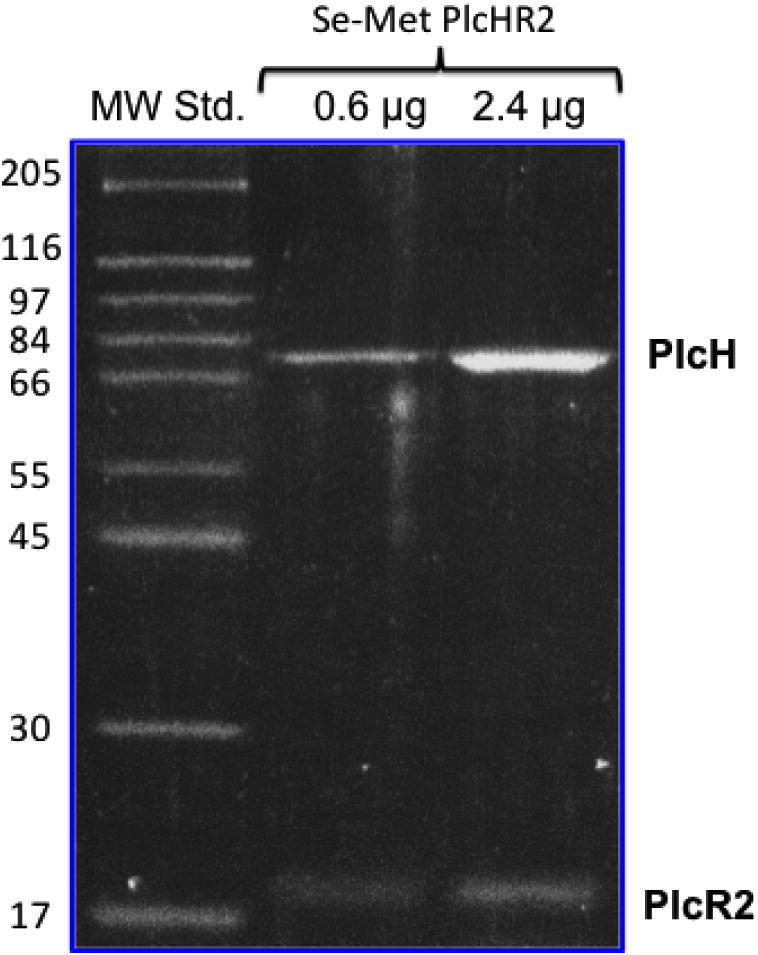
SDS–PAGE analysis of purified *Pseudomonas aeruginosa* PlcHR2 showing two distinct bands for the separated PlcH and the PlcR2 proteins.

Using *P. aeruginosa* as the natural host we were able to obtain mg amounts of pure and active PlcHR2. The single step purification resulted in >99% pure protein as judged from SDS PAGE analysis (Fig. 1) suitable for crystallization. l-selenomethionine substituted protein was expressed and purified in order to obtain crystals suitable to solve the crystallographic phase problem using MAD techniques [35]. The expression was thus successfully adapted by disrupting the inherent methionine pathway to incorporate l-selenomethionine from the medium. Although l-selenomethionine containing proteins have been expressed in the past in an auxotrophic *P. aeruginosa* strain [36], as well as *Pseudomonas fluorescence* [37], the method described here is tailor-made for the expression of extracellular virulence of *P. aeruginosa* that may be toxic for other expression hosts. ESI-MS analysis was used to verify that both components (PlcH and PlcR2) are present in the complex, and to assess the level of l-selenomethionine substitution. Given the size of the complex and the likely heterogeneity of substitution the molecular masses are not expected to be very accurate, however, peaks at 16,878 Da, 16,925 Da and 16,996 Da may correspond to the 1-, 2-, and 3-fold substitution of methionine by l-selonomethionine (mass difference 47 Da) in PlcHR2 (ESI-MS of the native protein 16,831 Da). In addition, there is a series of peaks with molecular weight of 78,706 Da, 78,745 Da and 78,800 Da, which may represent 7-, 8-, and 9-fold substitution with an expected molecular weight of 78,386 Da the unmodified PlcH. (Fig. S1 in Supplementary material).

### Crystallization

**Fig. 2:**
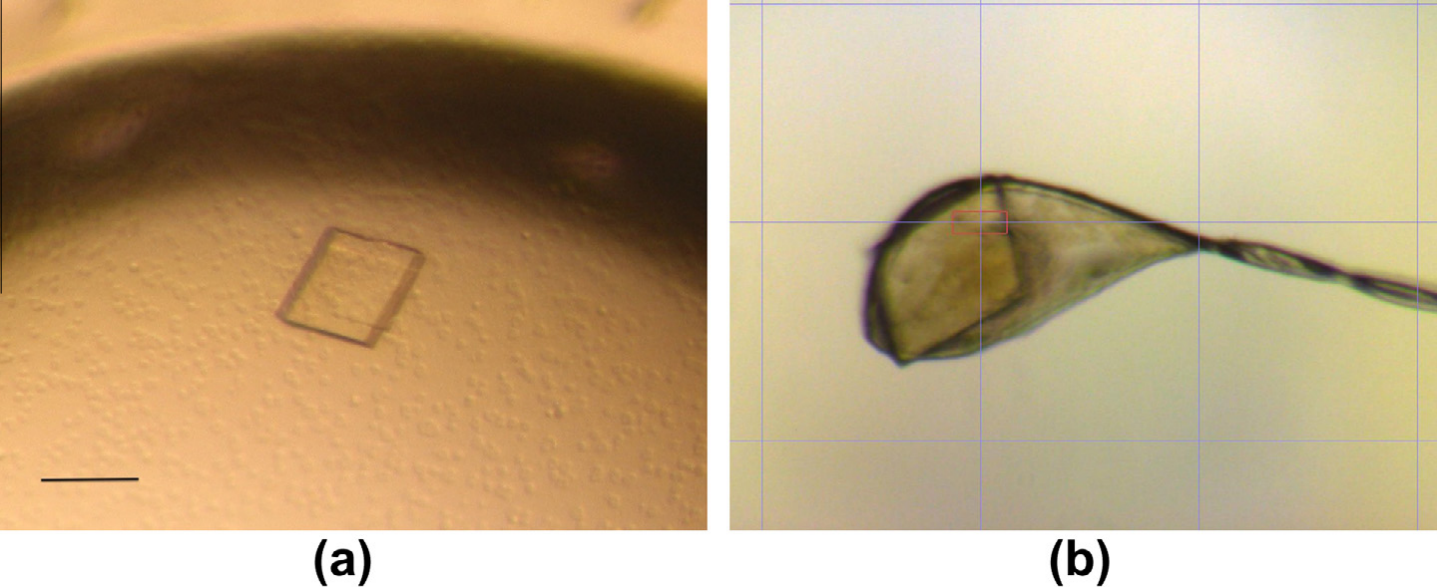
(a) Native crystals of PlcHR2 with approximate dimensions of 100 × 100 × 20 μm in the crystallization droplet. The scale bar represent approximately 100 μm.(b) Typical native crystal mounted in a nylon loop on the high-resolution diffractometer of beam line X10SA [29].

**Fig. 3:**
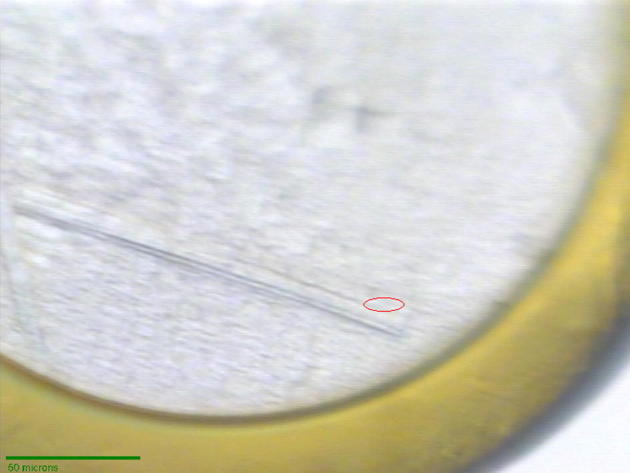
l-selenomethioine crystals mounted on the microdiffractometer MD2 at beam line X06SA. The red ellipsoid corresponds to the focused beam size of 25 × 6 μm (*Full width at half maximum*).

Native PlcHR2 crystals typically grew over a period of 1–2 months with maximum dimensions up to approximately 20 × 100 × 100 μm^3^ (Fig. 2a). Although the crystals were highly reproducible diffraction properties of crystals differed significantly not only from protein batch to batch but also between crystals from the same crystallization tray. l-selenomethionine substituted crystal grew typically for a period of 2 months to thin needles of maximal dimensions of 10 × 10 × 200 μm^3^. These crystals were hardly visible in sitting drop trays (data not shown) and proved to be extremely fragile (Fig. 3).

All attempts to increase diffraction quality of either native protein or l-selenomethionine substituted crystals by the various post-crystallization techniques including various stabilizing solutions [38], dehydration and crystal annealing [39–41] did not improve diffraction properties and more often than not quickly destroyed the crystals.

### Diffraction experiments

**Fig. 4:**
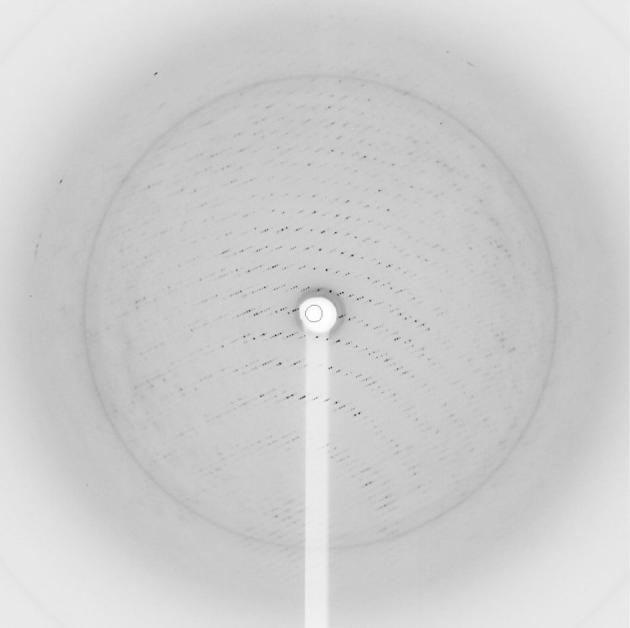
Diffraction pattern of a native crystal. Diffraction spots are clearly visible beyond the water ring at approximately 3.5 Å resolution.

Although the crystal diffracted to reasonable resolution on the SLS beam lines, radiation damage poses a major challenge [42]. Native PlcHR2 crystals typically showed noticeable signs of severe damage after less than 30 s of exposure. However, due to the relative large crystal size compared to beam focus complete data sets to medium resolution could be collected from one single crystal. The best diffraction was recorded to a Bragg spacing of about 3.5 Å, the diffraction data however are anisotropic with significantly higher mosaicity and lower resolution in one direction (Fig. 4).

**Fig. 5:**
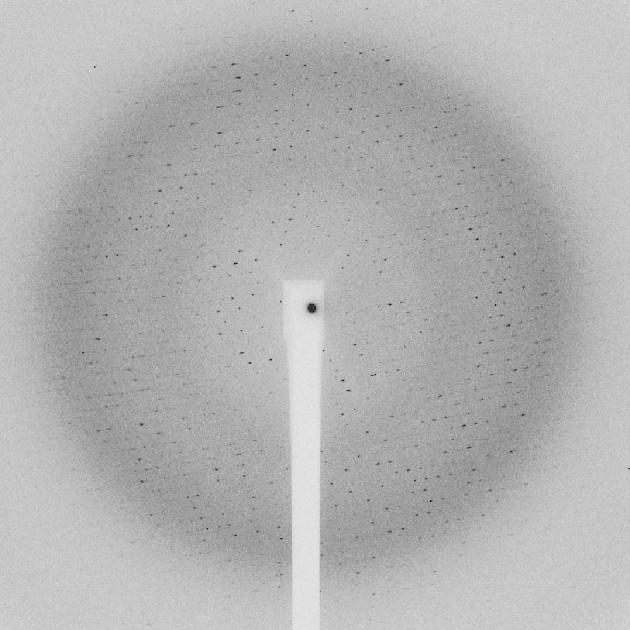
Diffraction pattern of a l-selenomethioine PlcHR2 crystal. The edge of the detector corresponds to approximately 2.5 Å resolution.

The l-selenomethionine crystals diffracted surprisingly well to a maximum Bragg spacing of 2.5 Å (Fig. 5). However, these crystals proved to be much more radiation sensitive. Due to the higher cross-section of selenium compared to sulfur in particular on the peak of the absorption edge, the crystals absorb more strongly and hence deteriorate much quicker. Clear signs of radiation damage were visible after 3 s of exposure with the full beam and hence a maximum of 20° of rotation only could be collected by careful attenuation. Beam attenuation of more than 50% led to an unusable weak diffraction pattern. In order to collect a complete data set the crystals were shifted manually by approximately 20 μm along the longest needle dimension after 20° of rotation. For each of these 20° sweeps the crystal position was optimized manually by diffraction-based alignment. This was of particular importance in the position where the loop was oriented in the plane of the on-axis microscope. The best diffraction data were obtained by this manual mode of *helical* data collection where the effects of radiation damage are mitigated by exposing a fresh part of the needle before the exposed part has already been completely destroyed. Helical data collection modes have been automated at the ESRF and the Diamond Light Source where the starting and end points for translation can be picked by the user and the optimal translation for a given oscillation range is calculated [43,44].

**Fig. 6:**
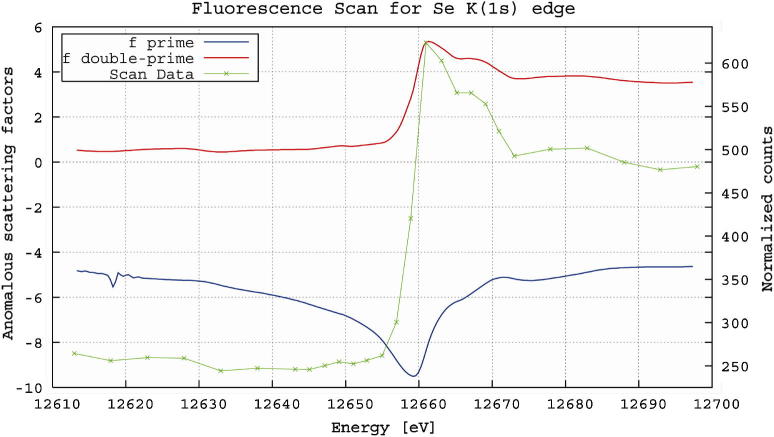
Typical fluorescence emission scan recorded from a l-selenomethionine PlcHR2 crystal on beam line X06SA. The values for f′ and f″ were calculated with CHOOCH [49].

The optimal wavelength for data collection on l-selenomethionine substituted crystals was determined by performing an X-ray emission fluorescence scan which clearly indicated the optimal energy for the collection of single-anomalous diffraction (SAD) phasing (Fig. 6). The scan also confirmed the incorporation of l-selenomethionine in place of methionine, at sufficient levels for structure solution. Data statistics from the highest resolution data sets are summarized in Table 1.

### Data analysis

The native protein crystallized in the orthorhombic space group C222_1_ with unit cell dimensions of *a* = 175.5, *b* = 196.4, and *c* = 325.3 Å. Given the limited diffraction properties it is reasonable to assume a higher than average solvent content which leads to between four and six PlcHR2 complexes in the asymmetric unit. Six complexes would translate to a solvent content of 50% with a Matthews factor of 2.46 Å^3^/Da whereas four molecules results in 67% solvent content and *V*_M_ = 3.7 Å^3^/Da [45,46]. The l-selenomethionine substituted protein crystallized in a much smaller unit cell with space group C2 and *a* = 158.4, *b* = 74.3, and *c* = 141.4 Å, β = 93.2°. The asymmetric unit is most likely to contain two independent complexes with a solvent content of 43% and *V*_M_ = 2.2 Å^3^/Da. The smaller unit-cell and the lower solvent content may explain the better diffraction properties of these crystals compared to the native protein.

### Molecular replacement

Structure solution attempts by molecular replacement were performed with a number of programs but mainly using PHASER [47] and Phenix/Rosetta [48]. Initial trials used the native data, but later when the higher resolution l-selenomethionine crystals became available the best data set as given in Table 1 was used. Several reduced search models based on the phosphodiesterase domain of the crystal structure of AcpA were constructed. Models were limited to the most similar part by removing all insertions and to the least flexible model where all flexible loops were also taken out. Models included multi-alanine structures where all residues that are different were changed to alanines and poly-alanine models. In addition, a second search fragment based on a very low similarity of the C-terminal *domain-of-unknown function* with the IG-like domain of human carcinoembryonic antigen cell adhesion molecule (pdb-code: 2DKS) was constructed and molecular replacement with two models was attempted. In spite of exhaustive efforts no convincing structure solution was obtained. It should be noted that given the low sequence identity of the search fragment constituting only about half of the scattering mass and the overall quality of the diffraction data this failure of molecular replacement trials had to be expected.

### Anomalous data and MAD phasing

In spite of the small crystals size (Fig. 3) the fluorescence emission scan depicted in Fig. 6 showed a clear signal and unambiguously confirmed the substitution of methionine by l-selenomethionine. However, due to the severe radiation damage it was not possible to collect diffraction data at more than one X-ray energy from a single crystal. Even the highest resolution and most complete data set collected from one crystal at the high-energy remote wavelength (summarized in Table 1) resulted in a relatively low overall *I*/*σ*(*I*) of 9.3 with an *R*_sym_ of 0.13. Considering the additional low crystallographic symmetry, which inherently results in low redundancy, it is no surprise that even at low resolution no useful anomalous signal was detected and the sub-structure of Se-positions could not be determined. Further data sets were collected from other crystals at the peak and inflection point determined by the fluorescence emission. The optimization of scaling procedures is currently underway in order to construct a useful 3-wavelength data set that will enable to solve the phase problem in the near future.

## Conclusions

In this paper we describe significant progress towards the crystal structure determination of the PlcHR2 complex from *P. aeruginosa*. Diffracting crystals of the native protein were obtained with detergents typically used for the crystallization of membrane proteins. However, since molecular replacement trials with native data to medium resolution failed, novel methods to over-express and purify l-selenomethionine substituted protein from its natural source *P. aeruginosa* were developed. These methods will have wider application for proteins that are less amenable for recombinant overexpression in *E. coli* due to toxicity and the typical inability of that organism to secret extracellular proteins. Further crystallization and the application of seeding techniques led to l-selenomethionine substituted micro-crystals that diffracted, albeit weakly to better than 2.5 Å resolution. Diffraction to this high resolution could only be recorded using the leading μ-focus beam lines highlighting once again the impact of 3rd generation synchrotron sources on macromolecular crystallography. Due to the limited crystal size and radiation sensitivity, the best crystals resulted in a data set to a resolution of 2.75 Å. Molecular replacement attempts have failed also for the higher resolution data set obtained from l-selenomethionine substituted crystals, presumably due to a combination of limited data quality and the lack of a sufficiently similar search model for structure solution. Successful structure solution will therefore require the application of improved data collection methods including optimized mounts for crystal positioning and the helical method where the crystal is rotated and shifted along the longest crystal dimension to minimize and smoothen the effects of radiation damage. Improved scaling procedures will help to merge data collected from different crystals in order to obtain a complete single-anomalous or if possible multiple anomalous data set required to solve the crystallographic phase problem.

## Figures and Tables

**Table 1:** Data collection and processing parameter.

	Native PlcHR2	Se-Met PlcHR2
Beam line	X10SA	X06SA
Wavelength [Å]	1.000	0.95370
Temperature [K]	100	100
Oscillation range [°]	1	1
No. of frames	180	170
Unit cell dimensions [Å], [°]	175.5, 196.4, 325.3	157.9, 75.4, 141.0, β = 93.2
Space group	C2221	C2
Max. resolution [Å]	4.0	2.75
No. of observed reflections	150 089	150 083
No. of unique reflections	45 739	43 080
Completeness (last shell)	95.6 (92.2)	99.7 (99.3)
*R*_sym_^a^(last shell)	0.103 (0.352)	0.130 (0.308)
*I*/*σ* (last shell)	8.3 (2.7)	9.3 (4.7)

a *R* = SUM(*I* − <*I*>)^2^/SUM(*I*^2^).
