# Corrigendum to “High-level over-expression, purification, and crystallization of a novel phospholipase C/sphingomyelinase from *Pseudomonas aeruginosa*” [Protein Expr. Purif. 90 (2013) 40–46]

**DOI:** 10.1016/j.pep.2014.08.008

**Published:** 2015-04

**Authors:** Daphné Truan, Adriana Vasil, Martin Stonehouse, Michael L. Vasil, Ehmke Pohl

**Affiliations:** a Swiss Light Source, Paul Scherrer Institute, 5232 Villigen, Switzerland; b Department of Microbiology, University of Colorado, School of Medicine, Anschutz Medical Center, Aurora, CO 80045, USA; c Department of Chemistry & School of Biological and Biomedical Sciences, Durham University, Durham DH1 3LE, UK

The authors regret that the reported microcrystals of l-selenomethionine PlcHR2 (space group C2 with *a* = 157.9 Å, *b* = 75.4 Å, *c* = 141.0 Å, *β* = 93.2) presented in Table 1 on page 44 have been solved and identified as the putative cysteine hydrolase YcaC from *Pseudomonas aeruginosa*. YcaC has presumably been co-purified as minor contaminant and crystallized from the l-selenomethioine protein preparations. Details of the structure determination and analysis of YcaC will be described elsewhere (Groftehauge, Truan, Vasil, Denny, Vasil & Pohl, 2014). The crystal structure analysis of native PlcHR2 (space group C222_1_, *a* = 175.5 Å, *b* = 196.4 Å, *c* = 325.3 Å) also reported in Table 1 is currently underway. The authors would like to apologize for any inconvenience caused.

