# Supplementary material for: High-level over-expression, purification, and crystallization of a novel phospholipase C/sphingomyelinase from Pseudomonas aeruginosa
Source: Protein Expr Purif. 2013 Jul;90(1):40–6. doi: 10.1016/j.pep.2012.11.005 (PMC3601568; doi:10.1016/j.pep.2012.11.005)

Figure S1a

Deconvoluted MS-ESI TOF of the L-selenomethionine substituted PlcHR2, (a) showing the entire mass range from 10000 to 90000 kDa with the peaks corresponding to PlcR2 clearly showing up around 16996 Da, and (b) a close-up around the expected molecular weight of PlcH.

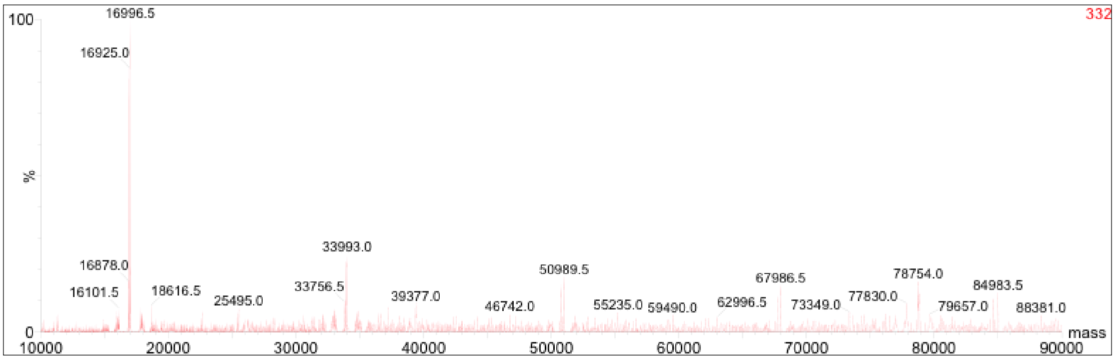

Figure S1b

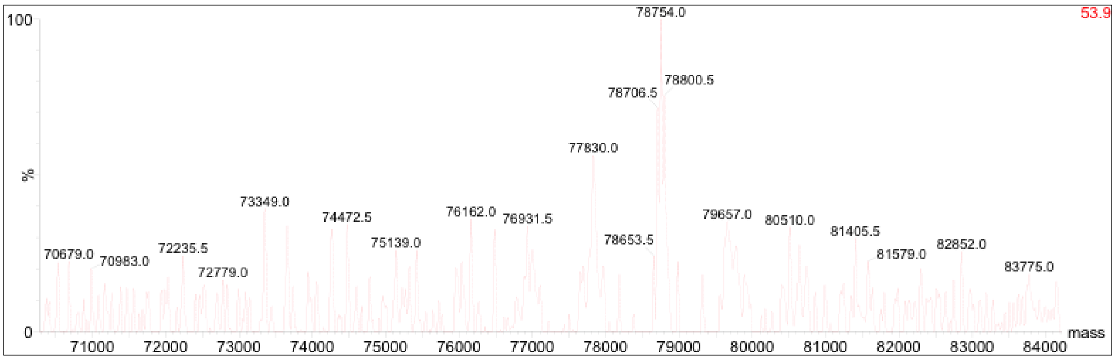

Supplement: Supplementary data 1 — Supplementary material. [file mmc1.pdf]
